# Changes in the Activity and Concentration of Superoxide Dismutase Isoenzymes (Cu/Zn SOD, MnSOD) in the Blood of Healthy Subjects and Patients with Acute Pancreatitis

**DOI:** 10.3390/antiox9100948

**Published:** 2020-10-01

**Authors:** Milena Ściskalska, Monika Ołdakowska, Grzegorz Marek, Halina Milnerowicz

**Affiliations:** 1 Department of Biomedical and Environmental Analyses, Faculty of Pharmacy, Wroclaw Medical University, 50-556 Wroclaw, Poland; monika.oldakowska@umed.wroc.pl; 2 Second Department of General and Oncological Surgery, Faculty of Medicine, Wroclaw Medical University, 50-556 Wroclaw, Poland; grzegorz.marek@umed.wroc.pl

**Keywords:** acute pancreatitis, SOD isoenzymes, SOD1, SOD2, SOD3, superoxide dismutase, rs2070424, single-nucleotide polymorphism, smoking, age

## Abstract

This study was aimed at evaluating the changes in the concentration and activity of all superoxide dismutase isoenzymes (SOD1, SOD2, SOD3) in the blood of patients with acute pancreatitis (AP) and healthy subjects, taking into account the extracellular (plasma) and intracellular (erythrocyte lysate) compartment. The relationships between the activity/concentration of SODs, metal concentration and the markers of inflammation were evaluated. To assess the pro/antioxidative imbalance, the malonyldialdehyde (MDA) concentration and the value of total antioxidant capacity (TAC) were measured. The impact of single-nucleotide polymorphism (SNP) in the SOD1 gene (rs2070424) on the activity/concentration of SOD1 as the main isoenzyme of the SOD family was also analyzed in this study. The SOD2 activity in erythrocytes was increased compared to plasma: 10-fold in the AP patient group and 5-fold in healthy subjects. The plasma of AP patients showed an increased SOD1 concentration and decreased SOD2 and SOD3 concentrations compared to healthy subjects. The Cu/Zn SOD (SOD1 + SOD3) concentration in plasma of AP patients was elevated compared to healthy subjects, but changes in plasma Cu/Zn SOD (SOD1 + SOD3) activity in the examined groups were not observed. An influence of SNP rs2070424 in the SOD1 gene on the total activity of SOD in AP patients (with AG genotype), accompanied by an increased IL-6 concentration, was observed. In oxidative stress conditions induced by inflammation, the participation of individual forms of plasma SOD isoenzymes in total antioxidative activity of SOD changed. A significant increase in the intracellular SOD1 concentration in plasma of AP patients proves the important role of this isoenzyme in the neutralization of oxidative stress induced by impaired Cu and Zn homeostasis. The presence of increased concentration of SOD2 in erythrocytes of healthy subjects and AP patients confirms the important function of this isoenzyme in the antioxidative defense.

## 1. Introduction

The superoxide dismutase (SOD) family plays an essential physiological role in mitigating the deleterious effects of oxygen-derived free radicals (ROS) [1]. It has been proven by immunohistochemistry that increased SOD expression reflects a defensive mechanism of pancreatic acinar cells against oxidative stress [2,3]. In mammals, there are three members of the SOD family with tightly regulated localization patterns: SOD1, SOD2 and SOD3 [1,4,5]. All SOD isoenzymes catalyze the dismutation of superoxide anion free radicals (O_2_^•−^) into molecular oxygen and hydrogen peroxide (H_2_O_2_) and decrease the O_2_^•−^ level which damages the cells at excessive concentrations [4,6,7].

Two copper/zinc superoxide dismutases (Cu/Zn SOD): SOD1 within the cytosol, mitochondrial intermembrane space, nucleus, lysosomes and peroxisomes and SOD3 (EcSOD), which is the predominant antioxidant enzyme secreted into the extracellular space, were found [1,4,8]. In these isoenzymes, Zn^2+^ ion plays a structural role, as it is required for efficient SOD folding and long-term stability, whereas Cu^2+^ in active sites catalyzes O_2_^•−^ dismutation by alternating between the reduced and oxidized states [9]. SOD1, in its homodimeric form, is a main intracellular isoenzyme of SODs. SOD3 is the extracellular superoxide dismutase which has unique characteristics and functions in the cellular signal transduction [1,10]. Recent studies have also shown that SOD3 can be localized in intracellular compartments in neutrophils and macrophages [11]. It is highly expressed in certain tissues, such as cardiovascular endothelium, placenta and lung, and moderately within kidney, pancreas and uterus, cartilage, skeletal muscle, adipose tissue, brain and eye [4,12]. This homotetrameric glycoprotein, due to the presence of a positively charged C-terminal region in its structure, is capable of binding proteoglycans contained in the cell membrane and constituents of the extracellular matrix [11].

The last SOD isoenzyme is SOD2; it is a manganese-containing superoxide dismutase (MnSOD) [13]. This enzyme is synthesized in the cytoplasm and translocated to the mitochondrial matrix; hence, it participates in cell protection against damages induced by superoxide anions produced by respiratory chain enzymes [7,14]. SOD2 is a homotetramer which is characterized by a longer circulating half-life compared to other SOD isoenzymes [13].

In the literature, there are reports about the role of SOD isoenzymes as therapeutic factors in inflammation [5,6,7,10]. It has been shown that there is an association between the single-nucleotide polymorphism (SNP) in the genes of SOD isoenzymes (especially SNP rs2070424 in the SOD1 gene) and inflammation and Cu concentration as a regulator of their activity [15,16]. However, the levels of individual SOD isoenzymes in patients with acute pancreatitis (AP) have not been studied so far. For this reason, determining the concentration and activity of SOD isoforms in the intracellular and extracellular compartments in AP patients appears to be important.

This study was aimed at evaluating the impact of acute pancreatitis on the changes in concentration and activity of all three superoxide dismutase isoenzymes: cytosolic SOD1, mitochondrial SOD2 and extracellular SOD3 in extracellular (plasma) and intracellular (erythrocyte lysate) compartments. The pro/antioxidative imbalance associated with AP was evaluated by the measurement of malonyldialdehyde (MDA) concentration and the value of total antioxidant capacity (TAC). To assess the intensity of inflammation, the concentrations of high-sensitivity C-reactive protein (hs-CRP) and IL-6 were determined. The impact of age, sex and tobacco smoke exposure on the activity of SOD isoenzymes and metal (Cu, Zn) homeostasis was also assessed in this study. The intensity of exposure to smoke xenobiotics was evaluated by determining the cotinine concentration. The impact of SNP in the SOD1 gene (rs2070424) on the activity/concentration of SOD1 as the main isoenzyme of the SOD family was also analyzed in this study.

## 2. Materials and Methods

### 2.1. Subjects

This study carried out on whole blood collected from patients with acute pancreatitis (*n* = 40) and healthy persons, whose age ranged from 30 to 70 years (*n* = 51). All subjects gave informed written consent for participation in the study. The study was conducted in accordance with the Declaration of Helsinki and approved by the Bioethics Committee of the Wrocław Medical University (No.: KB: 592/2013, KB: 529/2018 and KB: 215/2020). All participants filled out a questionnaire survey, containing questions about anthropometric data, use of pharmaceuticals, diet and lifestyle, including smoking and drinking alcohol.

The patients were hospitalized in the Second Department of General and Oncological Surgery, Wrocław Medical University. Patients were recruited for the study by clinicians on the basis of the criteria presented in Table 1. During hospitalization, the aforementioned patients were administered infusion fluids (crystalloid solution), the dosage of which depended on the degree of hydration or fluid overload. In all patients with diagnosed AP, a low-fat diet was applied. The criteria for exclusion from the study group included coexisting inflammatory diseases, liver diseases, diabetes and neoplastic diseases.

The recruitment of healthy subjects to participate in this study was carried out by physicians. Persons taking medications/dietary supplements and/or consuming excessive amounts of alcohol were excluded from this group.

The assessment of the degree of exposure to tobacco smoke xenobiotics was carried out on the basis of cotinine concentration (a nicotine metabolite). The participants were divided into the group of nonsmokers (cotinine concentration < 10 ng/mL) or smokers (cotinine concentration > 10 ng/mL).

### 2.2. Materials

Whole blood was collected from the ulnar vein using a Becton Dickinson Vacutainer system according to standard diagnostic procedures (in the morning, after an approximately 8-h break from eating and drinking). The study material included serum, plasma, erythrocyte lysate and DNA isolated from whole blood. In order to obtain serum, venous blood was collected in disposable test tubes with a clotting activator (Cat. No.: 368815, Becton Dickinson, Germany). The blood samples were left at 25 °C to complete thrombosis and then centrifuged (1200× *g*/20 min). Plasma and erythrocyte lysate were obtained by collecting whole blood in tubes containing heparin (Cat. No.: 368886, Becton Dickinson, Germany) and EDTA (Cat. No.: 367864, Becton Dickinson, Germany). Then the blood samples were centrifuged (2500× *g*/15 min) to separate plasma and buffy coat from erythrocytes. To obtain the erythrocyte lysate, erythrocytes were washed twice with an equal volume of ice-cold 0.9% NaCl and then lysed by adding ice-cold, double-distilled water in a ratio of 1:1.4. The obtained samples of serum, plasma and erythrocyte lysate were stored in closed tubes (Cat. No.: 0030102.002, Eppendorf, Germany) at −25 °C until analysis. The DNA was isolated from buffy coat, contained into EDTA tubes, and stored at −80 °C.

### 2.3. Methods

The serum cotinine concentration was measured using the commercial Cotinine ELISA test (Cat. No.: EIA-3242, DRG International, Inc., Mountain Avenue, NJ, USA) with an assay sensitivity of 1 ng/mL. The absorbance was measured at λ = 450 nm. This provided qualitative screening results for cotinine in human serum at a cut-off concentration of 10 ng/mL.

The concentration of intracellular copper-zinc superoxide dismutase (SOD1) was determined in plasma and erythrocyte lysate with a commercial kit (Cat. No.: BMS222, Thermofisher, Waktham, MA, USA) with monoclonal antihuman SOD1 antibodies (sensitivity: 0.04 ng/mL, intra-assay precision: 5.1%, *λ* = 450 nm). To measure the concentration of mitochondrial superoxide dismutase (SOD2) in plasma and erythrocyte lysate, a kit (Cat. No.: EKU07502, Biomatik, Wilmington, DE, USA) containing polyclonal antihuman SOD2 antibodies was used (sensitivity: 0.28 ng/mL, intra-assay precision: ˂10.0%, inter-assay precision: ˂12.0%, *λ* = 450 nm). The concentration of cytosolic superoxide dismutase (SOD3) in the plasma was determined with the kit (Cat. No.: EKU07504, Biomatik, Wilmington, DE, USA) using polyclonal antihuman SOD3 antibodies (sensitivity: 0.51 pg/mL, intra-assay precision: ˂10.0%, inter-assay precision: ˂12.0%, *λ* = 450 nm). The SOD concentrations in erythrocyte lysate were converted to milligrams of hemoglobin and expressed as ng/mg Hb.

The measurement of the activity of SOD isoenzymes in plasma and erythrocyte lysate was performed using a commercial kit with xanthine oxidase, hypoxanthine and tetrazole salt (Cat. No.: 706002, Cayman Chemical, Ann Arbor, MI, USA). The total SOD (SOD1 + SOD2 + SOD3) activity was measured with this kit, according to the manufacturer’s instructions (sensitivity: 0.005 U/mL, intra-assay precision: 3.2%, inter-assay precision: 3.7%, λ = 450 nm). To determine SOD2 (MnSOD) activity, the Cu/Zn SOD (SOD1 + SOD3) activity was inhibited by the addition of 3 mM potassium cyanide (KCN) (Cat. No. 1.04967.0100, Merck, Kenilworth, NJ, USA), as described earlier [17]. The absorbance of the samples was read at λ = 450 nm. Based on the difference between total SOD activity and SOD2 (MnSOD) activity, the Cu/ZnSOD activity (SOD1 + SOD3) was calculated. The activity of SOD isoenzymes in plasma was expressed as U/mL. One unit of SOD activity is defined as the amount of enzyme needed to dismutase 50% of available superoxide radicals. The activities of SOD in erythrocyte lysate were converted to grams of hemoglobin and expressed as U/g Hb. The activities of SOD in plasma and erythrocyte lysate were also converted to nanograms or micrograms of SOD as protein and expressed as U/ng or U/μg.

Metal (Cu, Zn) concentrations in serum were measured using flame atomic absorption spectrometry (FAAS) in an air-acetylene flame at a wavelength of λ = 324.8 nm on Solaar M6 (Solaar House, Cambridge, UK) in the Atomic Absorption Spectroscopy Laboratory of the Department and Clinic of Internal and Occupational Diseases, Wrocław Medical University (Wrocław, Poland). The reference solutions were Single-Element Copper (Zinc) Standard 1000 g/mL, certified by CPI International. Based on Cu and Zn concentrations, Cu/Zn ratio was calculated.

The IL-6 concentration in plasma was determined by a commercial test Human IL-6 DuoSet ELISA (Cat. No.: DY206-05, R&D Systems, Minneapolis, MN, USA) with the assay sensitivity 0.70 pg/mL (intra-assay precision: 2.0%, inter-assay precision: 3.8 %). The absorbance of the samples was measured at λ = 450 nm.

The high-sensitivity CRP (hs-CRP) concentration in serum was determined by the turbidimetric method with an hs-CRP test (Cat. No.: 31927, Biosystems, Barcelona, Spain) with an assay sensitivity of 0.06 mg/L (intra-assay precision: 1.5%, inter-assay precision: 3.0 %). The absorbance of the samples was measured at λ = 540 nm. The concentration of plasma MDA was measured with using a commercial test Lipid Peroxidation (MDA) Assay Kit (Cat. No.: MAK085-1KT, Sigma-Aldrich, Taufkirchen, Germany). This assay is based on the reaction of MDA with thiobarbituric acid to form a colorimetric product which is adequate for the MDA concentration in the sample. The absorbance of the sample was measured at λ = 532 nm.

The total antioxidant capacity (TAC) was measured in plasma with a commercial test, the OxiSelectTM Total Antioxidant Capacity (TAC) Assay Kit (Cat. No.: STA-360, Cell Biolabs, Inc., San Diego, CA, USA). In this method, uric acid was applied as a reference antioxidant. The TAC value was expressed as uric acid equivalents (UAE). Then, UAE values were converted to copper reducing equivalents (CRE) by multiplying the 2189 µM Cu^2+^/mM uric acid (1 mM of uric acid = 2189 µM Copper Reducing Equivalents). The CRE value of sample is relative to the total antioxidant capacity in the sample at λ = 490 nm.

#### Genotyping Analyses

The assessment of SNP rs2070424 in the SOD1 gene (National Center for Biotechnology Information, NCBI Reference Sequence: NG_008689.1) was performed using the PCR-RFLP (Polymerase Chain Reaction-Restriction Chain Fragments) technique. The process was carried out in three steps. Firstly, a fragment of the DNA sequence was amplified by the polymerase chain reaction (PCR). Primers were designed using computer simulations (in silico method). Based on the sequence of genes contained in the GenBank database (NCBI Reference Sequence: NG_008689.1), the sequence of the primer was determined using the Primer-Blast program (National Center for Biotechnology Information). The PCR reaction was performed with a reaction volume of 20 µL containing 100 pmol/L of primers (forward primer: AGTACTGTCAACCACTAGCA, reverse primer: CCAGTGTGCGGCCAATGATG), 2 µL extracted DNA, 12.8 µL PCR clean water and 4 µL Gold Hot Start PCR Mix (Cat. No.: SY550231, Syngen Biotech, Wrocław, Poland). The initial denaturation was performed at 95 °C for 15 min, and followed by 35 cycles within 40 s at 95 °C (denaturation), 35 s at 60 °C (annealing) and 45 s at 72 °C (elongation). A final elongation step was performed at 72 °C for 15 min. The PCR products were digested with MspI (10 U/μL) restriction enzyme (Cat. No.: ER0541, Thermo Fischer, Waltham, MA, USA), and the digested fragments were visualized in 3% agarose gel (Cat. No.: SY521011) with Green DNA Gel Stain (Cat. No.: SY521032, Syngen Biotech, Wrocław, Poland).

### 2.4. Statistical Analysis

The data were presented as mean ± SD and the 1st quartile (Q1), median and the 3rd quartile (Q3). In all cases, *p* < 0.05 was considered statistically significant. The categorical variables were analyzed by the χ2 test. The normality of variables was tested using the Shapiro-Wilk test. When the variables were normally distributed, the Student’s *t*-Test was used; otherwise, the nonparametric Mann-Whitney U test was used. The differences between many groups were analyzed using ANOVA and Tukey’s post hoc test. The assumption of the equality of variance was made by Levene’s test. Data analyses were performed using the Statistica software package, version 13.3 (Polish version; StatSoft, Cracow, Poland).

## 3. Results

### 3.1. The Antropometric Data in the Group of Healthy Subjects and AP Patients and Diagnostic Parameters of Acute Pancreatitis

The basic clinical parameters were presented taking into account exposure to tobacco smoke as the one of major factor contributing to acute pancreatitis (Table 2). In the group of smokers, the cotinine concentration (in both heathy subjects and AP patients) and GGT activity (in heathy subjects) was increased compared to nonsmokers. Additionally, GGT activity in the blood of AP patients was several times higher compared to healthy subjects in both groups, i.e., smokers and nonsmokers (Table 2).

### 3.2. The Concentrations and Activities of Intracellular and Extracellular SODs Isoenzymes, the Concentrations of Metals (Cu, Zn), Markers of Inflammation and Oxidative Stress in the Group of Healthy Subjects and AP Patients

In the erythrocyte lysate of AP patients, about a 3-fold increase was observed in the activities of total SOD (SOD1 + SOD2 + SOD3) and Cu/Zn SOD (SOD1 + SOD3) and nearly a 2-fold increase in SOD2 (MnSOD) activity compared to healthy subjects. It was observed that the SOD2 (MnSOD) concentration was more than 3-fold increased in erythrocytes of AP patients compared to healthy subjects (Table 3).

An analysis of the concentrations of SOD isoenzymes in extracellular environment revealed a 2-fold increase in SOD1 and decreased SOD2 and SOD3 in plasma of AP patients compared to healthy subjects. The plasma total SOD and Cu/Zn SOD (SOD1 + SOD3) concentrations in AP patients were also increased in comparison to healthy subjects. However, no differences in the activity of plasma Cu/Zn SOD (SOD1 + SOD3) were observed. A decrease in total SOD and SOD2 (MnSOD) activities in the plasma of AP patients was also found, compared to healthy subjects (Table 3).

A decrease in Zn and an increase in Cu concentrations were observed in the blood of AP patients compared to healthy subjects, thereby altering the Cu/Zn ratio. The markers of inflammation (concentration of IL-6 and hs-CRP) and oxidative stress (MDA concentration) were also significantly elevated in the blood of AP patients compared to healthy subjects. In the group of AP patients, an increased TAC level compared to healthy subjects was found (Table 3).

### 3.3. Results of Genotyping

**Figure 1:**
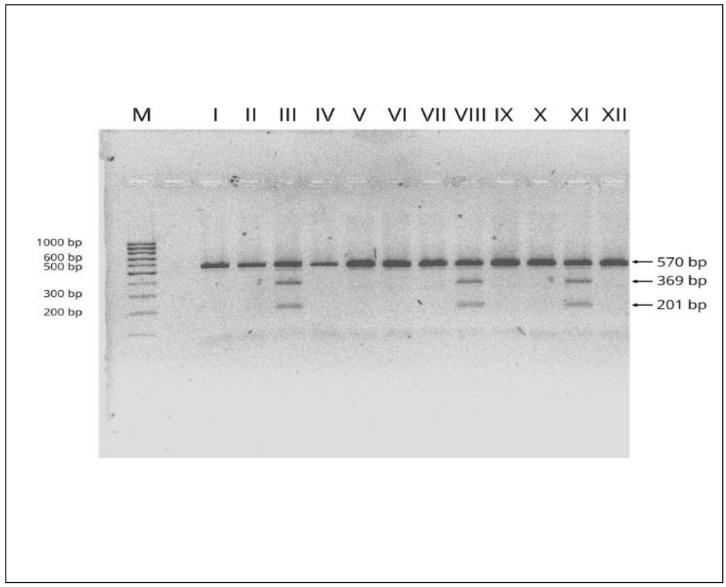
Examples of electropherograms used in the genotyping of rs2070424 in SOD1 gene (NCBI Reference Sequence: NG_008689.1). M—marker ladder (100–1000 bp); I, II, IV, V, VI, VII, IX, X, XII–AA genotype (570 bp fragment); III, VIII, XI–AG genotype (570, 369 and 201 bp fragments). Numbers are in base pair (bp).

We analyzed the concentrations and activities of SODs isoenzymes with respect to SNP rs2070424 in SOD1 gene (NCBI Reference Sequence: NG_008689.1) in the group of healthy subjects (aged 30–70) and AP patients. After the genomic DNA of the samples was amplified by PCR, the target 570-bp nucleotide sequences were seen in all samples. The identified genotypes were labelled according to the presence or absence of the enzyme restriction site for SNP rs2070424. The AA genotype is homozygous in the absence of the site (band at 570-bp), the AG is heterozygous in the presence and absence of the site (band at 570-, 369- and 201-bp) and the GG is homozygous in the presence of the restriction site (band at 369- and 201-bp). An example of an electropherogram showing the products after the restriction reaction in the group of AP patients and healthy subjects is presented in Figure 1.

In the group of AP patients, the AG genotype was detected in 14 cases (35% of AP patients group) and the AA genotype in 26 cases (65% of AP patients group). Among the healthy subjects, 25 cases of the AG genotype (41% of the healthy subjects group) and 36 cases with the AA genotype (59% of the healthy subjects group) were detected. In the examined samples, GG homozygous were not found. No differences in the frequency of occurrence of individual genotypes in the examined groups were shown (*x*^2^ = 0.5783, *p* = 0.4470).

### 3.4. Influence of SNP rs2070424 in SOD1 Gene on the Concentrations and Activities of Intracellular and Extracellular SODs Isoenzymes, the Concentrations of Metals (Cu, Zn), Markers of Inflammation and Oxidative Stress in the Group of Healthy Subjects Aged 30–70 and AP Patients

In the erythrocyte lysate of AP patients, increased activities of total SOD (SOD1 + SOD2 + SOD3) and Cu/Zn SOD (SOD1 + SOD3) were found compared to healthy subjects, in both the individuals with the AG and AA genotypes for SNP rs2070424 in SOD1 gene (Table 4).

In the group of healthy subjects, increased total SOD (SOD1 + SOD2 + SOD3) and Cu/Zn SOD (SOD1 + SOD3) activities were shown in subjects with the AG genotype compared to those with AA genotype for SNP rs2070424 in SOD1 gene (*p* = 0.0242, *p* = 0.0178 respectively) (Table 4).

In the group of AP patients, increased concentrations of total SOD (SOD1 + SOD2 + SOD3) and Cu/Zn SOD (SOD1 + SOD3) compared to healthy subjects were noted, in both individuals with the AG and the AA genotypes for the examined SNP. Interestingly, decreased activities of total SOD (SOD1 + SOD2 + SOD3) and Cu/Zn SOD (SOD1 + SOD3) were observed in the group of AP patients compared to healthy subjects only in individuals with the AG genotypes (Table 4).

An increased concentration of Cu in the blood of AP patients compared to healthy subjects was observed only in individuals with the AA genotypes for SNP rs2070424 in SOD1 gene. An increased value of the Cu/Zn ratio and a decrease in Zn concentration in AP patients compared to healthy subjects were found in individuals with the AG and AA genotypes. Similarly, increased concentrations of IL-6, hs-CRP, MDA and the level of TAC were shown in the individuals with the AG and AA genotypes for the examined SNP in the SOD1 gene (Table 4).

### 3.5. Selected Results of Correlations between the Concentration/Activity of SOD Isoenzymes and Other Parameters

The concentration/activity of total SODs (SOD1 + SOD2 + SOD3) and Cu/Zn SOD (SOD1 + SOD3) in the group of healthy subjects and AP patients in both plasma and erythrocyte lysates were correlated with other parameters.

In the group of healthy subjects a positive correlation was shown between the total SOD activity in plasma and Cu concentration. The activities of total SOD and SOD2 in plasma were negatively correlated with hs-CRP concentrations in this group. Additionally, it was observed that MDA concentration was negatively correlated with plasma SOD2 activity and total SOD activity in erythrocyte lysate (Table 5).

The strongest positive correlation was shown between the concentrations of Cu and IL-6 in the group of AP patients. In this group, the activity of Cu/Zn SOD in plasma was negatively correlated with MDA concentration, similar to plasma SOD2 concentration. Additionally, a negative correlation between the activity of Cu/Zn SOD in erythrocyte lysate and hs-CRP concentration or the value of the Cu/Zn index was noted (Table 5).

## 4. Discussion

The importance of the bioavailability of micronutrients in modulating the activity of SOD isoenzymes has been reported [18]. Additionally, SOD isoenzymes have been described as potential inhibitors of inflammation [6,19]. The anti-inflammatory role of SOD was also confirmed in our study by the negative correlation between SOD total activity in plasma and hs-CRP concentration in the group of healthy subjects.

The analysis of the SOD isoenzymes in erythrocyte lysate showed an increased total SOD activity in AP patients compared to healthy subjects. It was confirmed that SOD is the main enzyme component of the antioxidant system of erythrocytes, as shown in other studies [20]. SOD1 is primarily required for maintaining erythrocyte lifespan by suppressing oxidative stress. It was shown that SOD1 protects erythrocytes from the oxidative modification of proteins and lipids, resulting in anemia and the compensatory activation of erythropoiesis [21,22]. However, in our study, a decreased erythrocyte SOD1 concentration in AP patients was not observed. Increased Cu/Zn SOD (SOD1 + SOD3) activity in the erythrocyte lysate of AP patients compared to healthy subjects was noted. The measured activity of Cu/Zn SOD (SOD1 + SOD3) in erythrocytes reflects only SOD1 activity, because SOD3 is an extracellular isoenzyme. Therefore, the increased Cu/Zn SOD activity in the light of SOD1 activity could be the result of the erythrocyte exposure to oxidative stress over the course of AP.

In our study, we also analyzed the SOD2 level in erythrocytes. It is known that during normal erythropoiesis, red blood cells lose their mitochondria and other organelles as they mature [14]. It is widely recognized that SOD2—an isoenzyme exclusively located in mitochondria—is absent in mature erythrocytes [21,23,24]. Therefore, the presence of SOD2 in human erythrocytes has not been studied so far. Interestingly, it was shown in our studies that AP is associated with an increased SOD2 level in intracellular environment, measured as the concentration and activity of this isoenzymes in erythrocyte lysate. The changes in the concentration and activity SOD2 in erythrocytes can result from an impaired erythropoiesis in acute pancreatitis which is associated with a slightly progressing increase in the percentage of middle-aged reticulocytes, characterized by the presence of mitochondria [25]. Studies conducted on animal precursors of erythrocytes showed that SOD2 plays a role in the protection of erythrocytes from reduced deformability, increased hem-degradation products and an increased rate of hemoglobin oxidation [24]. This could partially explain the increased SOD2 level in erythrocyte lysate of AP patients compared to healthy subjects. On the other hand, an increase in the percentage of reticulocytes in the blood of AP patients is inadequate to the increase in SOD2 level. It has been reported that SOD2 expression is upregulated by superoxide radicals through the activation of the redox-sensitive transcription factors NF-kB and Nrf2 or inflammatory mediators, such as TNF and IL-1 [7,14]. It is possible that SOD2 in human erythrocytes exposed to intensive oxidative stress in the course of AP is not limited to mitochondria. The results described in this study clearly indicate the presence of SOD2 in erythrocytes in AP. This fact could cast new light on the role of SOD2 in red blood cells. The presence of SOD2 in the cytosol of erythrocytes may be a result of the migration of those mitochondrial isoenzymes from other cells circulating in plasma, such as leukocytes and platelets in the oxidative stress condition induced by inflammation. This could indicate an important role of SOD2 in the protection from oxygen oxidation in hemoglobin during the Fenton reaction. These results may emphasize the significance of SOD2 as an intracellular antioxidant in acute pancreatitis.

In our study, we also analyzed the concentration and activity of SOD isoenzymes in plasma as an extracellular environment. The results of our study showed a more than 3-fold decrease in SOD3 concentration in AP patients compared to healthy subjects. SOD3 is known as a predominant form of SODs in plasma [10]. In many studies, it has been shown that SOD3 is mainly expressed in endothelial cells [11,26,27], and its decrease was associated with vascular endothelium damage and the infiltration of inflammatory cells [5,10,28]. In our previous study [29], we demonstrated an increased concentration of endothelium damage markers in AP patients which could be associated with decreased SOD3 concentration in the plasma of these patients. Additionally, in other studies, it has been shown that the expression of SOD3 is depressed by TNF-α and IL-1α in cultured fibroblasts which are also engaged in inflammatory responses [30,31]. Decreased plasma SOD3 concentrations can be also the result of low bioavailability of Zn, which is reflected in the decreased Zn concentrations observed in the blood of AP patients compared to healthy subjects. A Zn deficiency in the course of pancreatitis has also been noted in another study [2]. These facts can be explained by lower SOD3 concentrations, as is a major extracellular SOD isoenzyme. Similarly, a decrease in SOD2 concentration accompanied by decreased SOD2 activity in plasma may be related to endothelium damage during AP, as reported in other studies [32,33,34]. This could suggest that SOD2 has a vital role in maintaining cellular and mitochondrial redox balance in venous endothelial cells to maintain their proper functioning [34,35].

A decrease in SOD2 and SOD3 concentrations results in decreased elimination of superoxide anions, increased oxidative stress and inflammation in the course of AP, which suggests an important role of these isoenzymes in the anti-inflammatory effect which was noted in other studies [7,10,20]. It is known that the role of SOD2 and SOD3 includes protecting against endothelium damage as one of pathomechanisms of AP development [6,7,19]. The decreased SOD3 and SOD2 concentrations shown in this study could be an important factor influencing the course of AP.

It was shown that the role of SOD3 in inflammation is not simply due to radical scavenging; it affects immune responses and cellular signal transduction [10]. In our previous study, the variable involvement of Cu/Zn SOD and disordered Cu and Zn homeostasis, depending on the progression of inflammatory processes in patients with pancreatitis, was shown [2]. Immunohistochemical localization in pancreatic tissue showed an intensified expression of Cu/Zn SOD in acinar cells and moderate reaction in the tail of the pancreas during pancreatitis [2,36]. The presence of those isoenzymes in the cystic fluids of the pancreas was also observed [2,36]. This could indicate that Cu/Zn SOD is an extremely important enzyme for the defense of this organ against oxidative stress and explain the two-fold increased SOD1 concentration in the plasma of AP patients compared to healthy subjects shown in this study. An increased SOD1 concentration in the plasma of AP patients resulted in an elevated concentration of total SODs and Cu/Zn SOD (SOD1 + SOD3), despite the decreased SOD2 and SOD3 concentrations. Interestingly, despite the changes in SOD1 and SOD3 concentrations, the activity of Cu/Zn SOD (SOD1 + SOD3) was unchanged. Therefore, this supports the view that an increase in SOD1 concentration is a regulatory mechanism which protects against a decrease in Cu/Zn SOD activity. Cytosolic SOD1 can be released into plasma form leukocytes and platelets as a result of damage to the cell membrane, reflected in increased MDA concentrations in AP patients. In this way, changes in the distribution of SOD1 from the intracellular to extracellular compartment during AP may complement a decrease in extracellular SOD3 concentrations. Our study showed that the Cu/Zn SOD activity during AP remained within a physiological range, but the involvement of SOD isoforms had changed. The maintenance of Cu/Zn SOD activity appears to be important for the protection of the cellular membrane against oxidative damage, which may confirm a negative correlation between plasma Cu/Zn SOD activity and MDA concentration in the blood of AP patients.

In our study, it was shown that the examined SNP in the SOD1 gene (NCBI Reference Sequence: NG_008689.1) did not affect plasma concentrations of SOD1. However, in AP patients with the AG genotype for SNP in the SOD1 gene, a decrease in the activity of SODs was observed. This could be associated with the Cu concentration in the blood of AP patients. In the group of AP patients with the AG genotype, an increased Cu concentration compared to heathy subjects was not observed, in contrast to the AP patients with the AA genotype. It was shown that Cu ions are essential to the catalytic activity of this enzyme [1,37]. An elevated Cu concentration observed in the blood of AP patients with the AA genotype could prevent the impairment of the activity of total SOD and Cu/Zn SOD, despite intensified oxidative stress. Additionally, in the group of AP patients with the AG genotype for SNP in the SOD1 gene, it was observed that decreased plasma total SOD and Cu/Zn SOD activity was accompanied by increased IL-6 concentrations (though this was statistically insignificant). This could confirm the role of Cu/Zn SOD activity (total SOD activities) in anti-inflammatory response. The association between SOD activities and inflammation was also confirmed by a positive correlation between the total activity of SOD isoforms and the CRP concentration in the group of healthy subjects.

In our study, the influence of age on decreased intracellular total SOD activity was shown (Table S1 in Supplementary Materials); this is consistent with other studies concerning animals and humans [38,39]. The changes in total SOD activity in elderly people may be caused by decreased SOD1 activity [38,39]. However, the increased total SOD activity in extracellular environment can be explained by increasing oxidative stress with aging, which underlines the important role of this enzyme in its neutralization [40]. Taking into consideration the aforementioned changes, analyses of SOD concentrations and activities in the course of AP presented in this study were carried out in relation to healthy subjects of similar age. However, the influences of sex and tobacco smoke exposure on total SOD activity were not observed (Tables S2–S5 in Supplementary Materials). Therefore, the aforementioned factors were not taken into account in the analysis of the levels of SOD isoenzymes in AP patients.

## 5. Conclusions

It was shown that in oxidative stress conditions induced by inflammation, the participation of individual forms of plasma SOD isoenzymes in the total antioxidative activity of SOD changes. A significant increase in intracellular SOD1 concentrations in the plasma of AP patients proved the important role of this isoenzyme in the neutralization of oxidative stress induced by impaired Cu and Zn homeostasis. The increased concentration of SOD2 in erythrocytes of healthy subjects and AP patients confirmed the important function of this isoenzyme in antioxidative defense. There is an influence of SNP rs2070424 in the SOD1 gene (NCBI Reference Sequence: NG_008689.1) on the total activity of SOD in AP patients (with AG genotype), which was accompanied by an increased IL-6 concentration.

## Figures and Tables

**Table 1:** Criteria for the inclusion the patients with AP for participation in the studies.

Diagnostic Methods	Inclusion Criteria
Medical history and clinical symptoms	a sudden onset of illness acute epigastric pain with tenderness on palpation
Laboratory diagnosis	at least three-fold increase in lipase or amylase activity in serum levels above the upper limit of the reference range increased of CRP concentration in the blood
Diagnostic imaging	Computed tomography with contrast enhancement patients with noncharacteristic abdominal pain for acute pancreatitis patients with amylase activity or serum lipase less than three times the upper limit of the reference range patients with uncertain diagnosis Magnetic resonance without contrast (gadolinium) patients with an allergy to contrast patients with renal failure

**Table 2:** The clinical characteristic of the study population.

Parameters	Healthy Subjects (*n* = 51)	
	Nonsmokers (*n* = 28) Mean ± SD	Smokers (*n* = 23) Mean ± SD
Age (years)	46.3 ± 9.0	46.3 ± 8.0
BMI (kg/m^2^)	22.7 ± 1.6	23.2 ± 2.0
Cotinine (ng/L)	0.7 ± 2.2	71.7 ± 47.2 *
Gender (women/men)	15/13	13/10
Glucose (mg/L)	86.3 ± 5.4	83.6 ± 18.2
Insulin (mU/mL)	6.8 ± 3.2	7.0 ± 5.0
Homa-IR GGT (U/L)	1.5 ± 0.7 32.1 ± 18.7	1.8 ± 1.3 45.2 ± 16.8 *
**Parameters**	**AP Patients (*n* = 40)**	
	**Nonsmokers (*n* = 17)** **Mean ± SD**	**Smokers (*n* = 23)** **Mean ± SD**
Age (years)	52.6 ± 18.0	46.2 ± 13.9
BMI (kg/m^2^)	27.6 ± 4.6	24.3 ± 4.3
Cotinine (ng/L)	1.2 ± 0.8	127.5 ± 51.4 *
Gender (women/men)	8/9	7/16
Lipase (U/L)	364.0 ± 313.9	430.7 ± 351.0
α-Amylase (U/L)	443.2 ± 333.8	369.6 ± 243.5
GGT (U/L)	283.7 ± 153.5 **	268.2 ± 159.8 **
Erythrocytes (10^12^/L)	4.8 ± 2.3	4.5 ± 0.8
Leukocytes (10^9^/L)	11.8 ± 4.3	12.7 ± 4.4
Hemoglobin (g/dL)	12.6 ± 2.3	13.6 ± 2.5
Hematocrit (%)	37.3 ± 5.6	40.1 ± 6.2
Trombocytes (10^9^/L)	297.2 ± 130.3	230.8 ± 133.7
Bilirubin (total) (mg/dL)	2.2. ± 1.8	1.8 ± 2.0
Alkaline phosphatase (U/L)	119.4 ± 67.7	113.2 ± 60.7
Glucose (mg/dL)	85.4 ± 17.5	89.3 ± 9.8

*p < 0.05 compared to nonsmokers, ** *p* < 0.05 compared to healthy subjects.

**Table 3:** The concentration and activities of SODs isoenzymes, the concentrations of metals (Cu, Zn), markers of inflammation and oxidative stress in the group of healthy subjects and AP patients.

Parameters in Erythrocyte Lysate	Healthy Subjects (*n* = 51)	AP Patients (*n* = 40)	*p*
SOD1 (ng/mg Hb)	5.8 ± 1.9 (4.5; 5.0; 6.8)	5.2 ± 2.1 (4.2; 4.9; 6.2)	0.2628
* SOD2 (ng/mg Hb)	1.9 ± 0.8 (1.4; 1.5; 2.4)	6.9 ± 3.3 (3.8; 5.3; 11.1)	<0.0001
* SODs (U/g Hb)	151.5 ± 55.4 (113.3; 141.9; 189.7)	419.5 ± 79.5 (371.2; 400.0; 472.1)	<0.0001
* Cu/Zn-SOD (U/ng SOD1 + SOD3)	143.4 ± 53.0 (106.1; 130.9; 163.6)	349.1 ± 90.9 (273.0; 337.5; 399.2)	<0.0001
* Mn-SOD (U/g Hb)	27.4 ± 13.4 (15.1; 28.2; 38.0)	49.8 ± 21.3 (31.5; 44.7; 75.3)	<0.0001
Mn-SOD (U/µg SOD2)	15.2 ± 3.6 (12.2; 13.4; 16.5)	7.8 ± 4.5 (12.3; 12.9; 13.4)	0.5697
**Parameters in plasma**	**Healthy subjects** **(*n* = 51)**	**AP patients** **(*n* = 40)**	***p***
* SOD1 (ng/mL)	32.5 ± 6.5 (27.8; 32.7; 35.8)	79.0 ± 27.5 (66.9; 79.4; 92.5)	<0.0001
* SOD2 (ng/mL)	27.7 ± 8.8 (21.0; 24.3; 32.9)	12.0 ± 3.2 (9.8; 10.9; 13.6)	<0.0001
* SOD3 (ng/mL)	25.8 ± 9.9 (17.8; 28.8; 30.6)	8.8 ± 2.8 (7.0; 9.1; 9.8)	<0.0001
* SOD1 + SOD2 + SOD3 (ng/mL)	66.4 ± 24.9 (52.6; 60.3; 80.9)	100.8 ± 60.2 (51.2; 94.8; 125.0)	0.0318
* SOD1 + SOD3 (ng/mL)	42.2 ± 21.9 (30.0; 32.6; 41.9)	92.5 ± 58.8 (49.9; 90.4; 114.2)	0.0057
* SODs (U/mL)	10.1 ± 1.4 (9.0; 10.2; 11.1)	8.9 ± 2.7 (7.2; 8.3; 10.5)	0.0067
Cu/Zn-SOD (U/mL)	5.3 ± 1.5 (4.2; 5.3; 6.6)	4.9 ± 1.8 (3.3; 4.6; 6.4)	0.2351
* Mn-SOD (U/mL)	5.3 ± 1.5 (4.2: 4.9; 6.4)	4.3 ± 1.4 (3.5; 4.0; 5.2)	0.0036
Cu/Zn-SOD (U/ng SOD1 + SOD3)	0.2 ± 0.1 (0.1; 0.2; 0.3)	0.1 ± 0.1 (0.0; 0.1; 5.5)	0.1779
SODs (U/ng SOD1 + SOD2 + SOD3)	0.2 ± 0.1 (0.1; 0.2; 0.3)	0.1 ± 0.0 (0.1; 0.1; 0.2)	0.7010
Mn-SOD (U/ng SOD2)	0.2 ± 0.0 (0.1; 0.2; 0.3)	0.4 ± 0.1 (0.2; 0.3; 0.5)	0.0711
* Cu (µg/L)	1037.4 ± 147.1 (952.4; 1027.5; 1107.0)	1120.1 ± 157.1 (1000.0; 1098.4; 1231.0)	0.0286
* Zn (µg/L)	938.3 ± 133.4 (846.1; 923.9: 1019.0)	625.7 ± 190.6 (480.5; 600.0; 786.7)	<0.0001
* Cu/Zn	1.1 ± 0.2 (1.0; 1.1; 1.2)	1.7 ± 0.4 (1.4; 1.7; 2.1)	<0.0001
* IL-6 (pg/mL) * hs-CRP (mg/L)	0.5 ± 0.4 (0.2; 0.3; 0.7) 0.5 ± 0.3 (0.4; 0.5; 0.9)	56.8 ± 29.9 (31.9; 49.7; 83.8) 101.2 ± 66.4 (39.2; 122.8; 138.5)	<0.0001 <0.0001
* MDA (nmol/µL)	0.8 ± 0.7 (0.3; 0.6; 1.2)	2.4 ± 0.7 (1.9; 2.3; 2.8)	<0.0001
* TAC (µM CRE)	44.7 ± 44.0 (14.2; 28.8; 53.5)	895.3 ± 391.2 (700.0; 783.5; 1254.6)	<0.0001

Values shown as mean ± SD (1st quartile, median, 3rd quartile), * significant difference (*p* < 0.05) between examined groups.

**Table 4:** Concentration and Activities of SODs Isoenzymes, Concentrations of Metals (Cu, Zn), Markers of Inflammation and Oxidative Stress in the Group of Healthy Subjects and AP Patients Divided in Terms of Genotype for SNP rs2070424 in SOD1 Gene (NCBI Reference Sequence: NG_008689.1).

Parameter in Erythrocyte Lysate	Genotype	Healthy Subjects (*n* = 51)	AP Patients (*n* = 40)	*p*
SOD1 (ng/mg Hb)	AG	5.1 ± 0.6 (4.6; 5.0; 5.7)	4.7 ± 1.9 (4.0; 5.0; 5.8)	0.9494
	AA	6.1 ± 2.2 (4.5; 5.0; 8.0)	5.1 ± 1.8 (4.3; 4.9; 6.2)	0.0734
*^,^** **SODs (U/g Hb)**	AG	148.7 ± 54.8 (126.5; 141.9; 165.3)	425.1 ± 63.4 (371.2; 440.2; 472.5)	<0.0001
	AA	153.3 ± 56.8 (111.3; 141.4; 194.7)	429.4 ± 87.1 (377.5; 398.5; 476.4)	<0.0001
*^,^** **Cu/Zn-SOD (U/g Hb)**	AG	143.9 ± 55.1 (110.4; 126.9; 160.7)	364.6 ± 99.8 (297.7; 347.2; 433.0)	<0.0001
	AA	143.0 ± 52.5 (100.1; 136.4; 165.7)	330.8 ± 83.3 (268.9; 309.6; 379.9)	<0.0001
**Parameter in plasma**		**Healthy subjects** **(*n* = 51)**	**AP patients** **(*n* = 40)**	***p***
*^,^** SOD1 (ng/mL)	AG	30.4 ± 5.5 (25.6; 29.2; 34.6)	86.7 ± 15.0 (74.4; 89.1; 97.8)	0.0012
	AA	33.6 ± 6.8 (28.7; 33.6; 37.2)	79.9 ± 30.2 (68.6; 79.0; 90.4)	0.0013
SOD1 + SOD2 + SOD3 [ng/mL]	AG	64.0 ± 32.2 (41.7; 53.4; 85.0)	88.2 ± 54.3 (26.9; 105.0; 118.9)	0.3692
	AA	68.6 ± 14.6 (55.8; 65.6; 80.9)	104.9 ± 60.5 (62.2; 86.0; 127.8)	0.0881
*^,^** SOD1 + SOD3 (ng/mL)	AG	48.0 ± 29.1 (30.3; 33.1; 61.1)	78.8 ± 39.0 (78.5; 91.6; 100.6)	0.0010
	AA	36.4 ± 10.4 (29.7; 32.4; 39.1)	66.9 ± 17.0 (40.0; 66.1; 95.9)	0.0144
*^,^*** SODs (U/mL)	AG	10.6 ± 1.2 (9.6; 10.6; 11.3)	8.3 ± 2.4 (7.1; 8.2; 8.4)	<0.0001
	AA	9.7 ± 1.4 (8.9; 9.7; 10.9)	9.3 ± 2.9 (7.3; 8.9; 11.2)	0.9997
*^,^*** Cu/Zn-SOD (U/mL)	AG	5.9 ± 1.4 (5.0; 6.1; 6.9)	5.0 ± 2.0 (4.0; 4.2; 6.4)	0.0316
	AA	4.8 ± 1.5 (3.7; 4.7; 5.6)	4.9 ± 1.7 (3.5; 5.0; 6.4)	0.9997
SODs (U/ng SOD1 + SOD2 + SOD3)	AG	0.2 ± 0.1 (0.1; 0.2; 0.3)	0.1 ± 0.0 (0.1; 0.1; 0.2)	0.4437
	AA	0.2 ± 0.1 (0.1; 0.2; 0.3)	0.1 ± 0.1 (0.1; 0.1; 0.2)	0.1559
Cu/Zn-SOD (U/ng SOD1 + SOD3)	AG	0.2 ± 0.1 (0.1; 0.2; 0.3)	0.1 ± 0.1 (0.0; 0.1; 0.1)	0.5831
	AA	0.2 ± 0.1 (0.1; 0.2; 0.3)	0.1 ± 0.1 (0.0; 0.1; 0.1)	0.1878
** Cu (µg/L)	AG	1063.9 ± 135.1 (986.9; 1054.4; 1131.7)	1091.6 ± 205.2 (971.7; 1010.8; 1206.7)	0.0866
	AA	1020.5 ± 154.3 (920.7; 1015.1; 1093.8)	1120.2 ± 128.7 (1014.0; 1126.3; 1218.8)	<0.0001
*^,^** Zn (µg/L)	AG	965.0 ± 153.6 (860.4; 923.8; 1043.3)	663.1 ± 208.7 (517.2; 609.6; 811.5)	<0.0001
	AA	919.4 ± 116.5 (838.5; 924.0; 1011.9)	597.5 ± 168.8 (476.2; 559.0; 728.8)	<0.0001
*^,^** Cu/Zn	AG	1.1 ± 0.2 (1.0; 1.1; 1.2)	1.8 ± 0.4 (1.6; 1.8; 2.1)	<0.0001
	AA	1.1 ± 0.2 (1.0; 1.1; 1.2)	1.7 ± 0.5 (1.4; 1.6; 1.9)	<0.0001
*^,^** IL-6 (pg/mL)	AG	0.5 ± 0.4 (0.2; 0.4; 0.7)	70.0 ± 28.0 (47.0; 68.6; 92.9)	<0.0001
	AA	0.5 ± 0.4 (0.1; 0.3; 0.6)	48.5 ± 33.7 (30.9; 31.9; 74.6)	<0.0001
*^,^** MDA (nmol/µL)	AG	0.9 ± 0.7 (0.3; 0.6; 1.3)	2.2 ± 0.5 (1.9; 2.1; 2.5)	<0.0001
	AA	0.8 ± 0.6 (0.3; 0.6; 1.0)	2.5 ± 0.8 (2.1; 2.4; 2.8)	<0.0001
*^,^** hs-CRP (mg/L)	AG	0.6 ± 0.3 (0.4; 0.5; 0.9)	131.5 ± 55.8 (139.5; 134.7; 157.0)	<0.0001
	AA	0.7 ± 0.3 (0.4; 0.6; 0.9)	111.1 ± 67.0 (58.3; 128.2; 158.3)	<0.0001
*^,^** TAC (µM CRE)	AG	52.3 ± 45.7 (10.5; 28.9; 51.7)	847.9 ± 334.8 (793.4; 771.8; 988.4)	<0.0001
	AA	40.1 ± 31.0 (15.1; 28.6; 55.4)	960.3 ± 405.1 (700.0; 800.7; 1279.3)	<0.0001

Values shown as mean ± SD (1st quartile, median, 3rd quartile), * significant difference (*p* < 0.05) between the group of healthy subjects and AP patients with the AG genotype, ** significant difference (*p* < 0.05) between the group of healthy subjects and AP patients with the AA genotype, *** significant difference (*p* < 0.05) between individuals with the AG genotype and AA genotype in the group of healthy subjects.

**Table 5:** Correlation coefficients for the group of healthy subjects and AP patients.

Variables	*r*	*p*
Healthy Subjects		
Activity of SODs (plasma)—hs-CRP	−0.3437	0.0373
Activity of SODs (plasma)—Cu	0.3809	0.0117
Activity of SODs (erythrocytes)—MDA	−0.3523	0.0239
Activity of SOD2 (plasma)—hs-CRP	−0.4422	0.0127
Activity of SOD2 (plasma)—MDA	−0.4161	0.0050
**Patients with Acute Pancreatitis**		
Cu—IL-6	0.7518	0.0030
Activity of Cu/Zn SOD (plasma)—MDA	−0.4157	0.0434
Activity of Cu/Zn SOD (erythrocytes)—Cu/Zn	−0.3908	0.0046
Activity of Cu/Zn SOD (erythrocytes)—hs-CRP	−0.4871	0.0074
Concentration of SOD2 (plasma)—MDA	−0.3554	0.0361

## Supplementary Materials

The following are available online at https://www.mdpi.com/2076-3921/9/10/948/s1, Table S1: The activity of total SOD, the concentrations of metals (Cu, Zn), MDA and hs-CRP in the blood of healthy subjects aged 20–30 and 30–70, Table S2: The activity of total SOD, the concentrations of metals (Cu, Zn), MDA and hs-CRP in the blood of individuals in 20–30 years old divided in terms of gender, Table S3: The activity of total SOD, the concentrations of metals (Cu, Zn), MDA and hs-CRP in the blood of individuals in 30–70 years old divided in terms of gender, Table S4: The activity of total SOD, the concentrations of metals (Cu, Zn), MDA and hs-CRP in the blood of individuals in aged 20–30 divided in terms of the exposure to tobacco smoke xenobiotics, Table S5: The activity of total SOD, the concentrations of metals (Cu, Zn), MDA and hs-CRP in the blood of individuals in aged 30–70 divided in terms of the exposure to tobacco smoke xenobiotics.
