# Supplementary material for: Changes in the Activity and Concentration of Superoxide Dismutase Isoenzymes (Cu/Zn SOD, MnSOD) in the Blood of Healthy Subjects and Patients with Acute Pancreatitis
Source: Antioxidants (Basel). 2020 Oct 1;9(10):948. doi: 10.3390/antiox9100948 (PMC7601220; doi:10.3390/antiox9100948)
Supplement: Supplementary file 1 [file antioxidants-09-00948-s001.pdf]

1.1. The total SOD Activity, Concentrations of Metals (Cu, Zn), hs-CRP and MDA in Healthy Subjects Divided in Terms of Age, Gender and Exposure to Tobacco Smoke Xenobiotics

In plasma of healthy subjects aged 30–70 was shown a 2-fold increase in total SOD activity (SOD1 + SOD2 + SOD3) compared to individuals in 20–30 years old. The activity of this antioxidant in erythrocyte lysate was more than 4-fold decreased in the group of subjects aged 30–70 compared to subjects in 20–30 years old. It was also shown that Cu and Zn concentrations were increased in the group of heathy subjects aged 30–70 compared to individuals in 20–30 years old (Table 1).

In the group of healthy subject aged 20–30, a lower Zn concentration and increased the value of Cu/Zn ratio in the blood of men compared to women were shown (Table 2). A similar changes in above mentioned parameters were observed in the group of healthy subjects in 30–70 years old (Table 3).

An increased the concentration of Cu and Zn in the blood of smoking of healthy subjects in 20–30 years old compared to non-smokers were noted (Table 4). No differences in the activity of total SOD, the concentrations of metals (Cu, Zn), MDA and hs-CRP between smokers and non-smokers in aged 30–70 were shown (Table 5).

Table S1. The activity of total SOD, the concentrations of metals (Cu, Zn), MDA and hs-CRP in the blood of healthy subjects aged 20–30 and 30–70.

Parameter	Healthy Subjects (20–30 years old) (n = 92)	Healthy Subjects (30–70 years old) (n = 51)	p
*** SODs (U/mL)**	5.9 ± 0.5<br>(5.6; **5.8**; 6.2)	10.1 ± 1.4<br>(9.0; **10.2**; 11.1)	**<0.0001**
*** SODs (U/g Hb) [1]**	646.1 ± 171.6<br>(543.5; **644.1**; 741.5)	151.5 ± 55.4<br>(113.3; **141.9**; 189.7)	**<0.0001**
*** Cu (µg/L)**	971.7 ± 133.1<br>(871.0; **965.0**; 1052.0)	1037.4 ± 147.1<br>(952.4; **1027.5**; 1107.0)	**0.0188**
*** Zn (µg/L)**	860.4 ± 115.1<br>(771.0; **847.5**; 951.0)	938.3 ± 133.4<br>(846.1; **923.9**; 1019.0)	**0.0011**
Cu/Zn	1.1 ± 0.3<br>(0.9; **1.07**; 1.3)	1.1 ± 0.2<br>(1.0; **1.1**; 1.2)	0.2566
MDA (nmol/µL)	1.0 ± 0.6<br>(0.6; **1.0**; 1.4)	0.8 ± 0.7<br>(0.3; **0.6**; 1.2)	0.1388
hs-CRP (mg/L)	0.4 ± 0.2<br>(0.1; **0.2**; 0.4)	0.5 ± 0.3<br>(0.4; **0.5**; 0.9)	0.3313

Values shown as (1st quartile, median, 3rd quartile), * significant difference (p < 0.05) between examined groups, [1] Total SOD activity measured in erythrocyte lysate.

Table S2. The activity of total SOD, the concentrations of metals (Cu, Zn), MDA and hs-CRP in the blood of individuals in 20–30 years old divided in terms of gender.

Parameter	Women (n = 62)	Men (n = 30)	p
SODs (U/mL)	5.9 ± 0.4<br>(5.2; **5.8**; 6.1)	6.1 ± 0.6<br>(5.7; **6.0**; 6.6)	0.1025
SODs (U/g Hb) [1]	652.9 ± 183.3<br>(550.9; **644.1**; 741.5)	626.4 ± 137.1<br>(538.7; **636.1**; 730.9)	0.5277
Cu (μg/L)	988.7 ± 131.2<br>(896.0; **975.5**; 1072.0)	918.2 ± 128.3<br>(816.0; **900.5**; 1005.0)	0.0944
*** Zn (μg/L)**	842.4 ± 100.6<br>(771.0; **838.0**; 920.1)	918.0 ± 129.0<br>(830.0; **903.0**; 992.0)	**0.0119**
*** Cu/Zn**	1.1 ± 0.2<br>(1.0; **1.1**; 1.3)	0.9 ± 0.1<br>(0.8: **0.9**; 1.0)	**0.0005**
MDA (nmol/μL)	0.9 ± 0.5<br>(0.6; **0.8**; 1.2)	1.1 ± 0.6<br>(0.5; **1.1**; 1.5)	0.2647
hs-CRP (mg/L)	0.4 ± 0.2<br>(0.1; **0.2**; 0.5)	0.3 ± 0.2<br>(0.1; **0.3**; 0.4)	0.5741

Values shown as (1st quartile, median, 3rd quartile), * significant difference ($p < 0.05$) between examined groups, [1] Total SOD activity measured in erythrocyte lysate.

Table S3. The activity of total SOD, the concentrations of metals (Cu, Zn), MDA and hs-CRP in the blood of individuals in 30–70 years old divided in terms of gender.

Parameter	Women (n = 62)	Men (n = 30)	p
SODs (U/mL)	10.3 ± 1.5<br>(9.0; **10.4**; 11.4)	9.7 ± 0.9<br>(9.0; **9.7**;10.4)	0.2214
SODs (U/g Hb) [1]	158.7 ± 54.3<br>(114.3; **144.2**; 190.9)	140.5 ± 57.0<br>(96.0; **133.1**; 165.3)	0.2965
Cu (μg/L)	1056.8 ± 126.1<br>(977.7; **1038.8**; 1110.7)	1001.2 ± 179.0<br>(838.4; **991.9**; 1089.5)	0.2260
*** Zn (μg/L)**	911.2 ± 142.6<br>(838.9; **898.7**; 992.7)	1000.2 ± 84.7<br>(970.9; **1014.3**; 1043.3)	**0.0358**
*** Cu/Zn**	1.2 ± 0.2<br>(1.0; **1.2**; 1.3)	1.0 ± 0.1<br>(0.9; **1.0**; 1.1)	**0.0012**
MDA (nmol/μL)	0.9 ± 0.6<br>(0.4; **0.7**; 1.3)	1.0 ± 0.6<br>(0.4; **1.1**; 1.5)	0.1407
hs-CRP (mg/L)	0.5 ± 0.3<br>(0.2; **0.3**; 0.5)	0.4 ± 0.3<br>(0.2; **0.4**; 0.5)	0.4581

Values shown as (1st quartile, median, 3rd quartile), * significant difference ($p < 0.05$) between examined groups, [1] Total SOD activity measured in erythrocyte lysate.

Table S4. The activity of total SOD, the concentrations of metals (Cu, Zn), MDA and hs-CRP in the blood of individuals in aged 20–30 divided in terms of the exposure to tobacco smoke xenobiotics.

**Parameter**	**Non-Smokers (*n* = 59)**	**Smokers (*n* = 23)**	***p***
SODs (U/mL)	5.9 ± 0.5 (5.6; **5.8**; 6.1)	6.0 ± 0.45 (5.6; **6.0**; 6.4)	0.2999
SODs (U/g Hb) [1]	655.9 ± 180.1 (547.2; **648.7**; 743.2)	473.1 (473.1; **473.1**; 473.1)	0.7485
*** Cu (μg/L)**	935.3 ± 132.4 (848.0; **905.5**; 1010.0)	1040.8 ± 106.4 (960.5; **998.5**; 1124.0)	**0.0032**
*** Zn (μg/L)**	839.7 ± 106.3 (755.0; **829.0**; 919.0)	936.3 ± 116.9 (848.0; **920.0**; 988.0)	**0.0033**
Cu/Zn	1.1 ± 0.3 (0.9; **1.1**; 1.2)	1.1 ± 0.2 (0.9; **1.0**; 1.3)	0.3727
MDA (nmol/μL)	0.9 ± 0.5 (0.4; **0.9**; 1.3)	1.0 ± 0.6 (0.7; **0.8**; 1.5)	0.3729
hs-CRP (mg/L)	0.4 ± 0.3 (0.1; **0.3**; 0.4)	0.3 ± 0.2 (0.1; **0.3**; 0.5)	0.9782

Values shown as (1st quartile, median, 3rd quartile), * significant difference ($p < 0.05$) between examined groups, [1] Total SOD activity measured in erythrocyte lysate.

Table S5. The activity of total SOD, the concentrations of metals (Cu, Zn), MDA and hs-CRP in the blood of individuals in aged 30–70 divided in terms of the exposure to tobacco smoke xenobiotics.

**Parameter**	**Non-smokers (*n* = 28)**	**Smokers (*n* = 23)**	***p***
SODs (U/mL)	10.1 ± 1.3 (9.1; **10.3**; 1.1)	10.1 ± 1.5 (8.9; **10.1**; 11.3)	0.8814
SODs (U/g Hb) [1]	157.4 ± 60.0 (114.3; **142.2**; 194.7)	141.4 ± 48.8 (103.7; **140.0**; 176.6)	0.3504
Cu (μg/L)	1006.2 ± 128.1 (919.3; **994.6**; 1107.0)	1068.7 ± 160.7 (989.0; **1035.5**; 1210)	0.1520
Zn (μg/L)	936.4 ± 129.0 (840.0; **914.0**; 1010.1)	940.7 ± 142.7 (852.5; **957.2**; 1010.5)	0.9159
Cu/Zn	1.1 ± 0.2 (1.0; **1.1**; 1.2)	1.1 ± 0.2 (1.0; **1.1**; 1.2)	0.8018
MDA (nmol/μL)	0.9 ± 0.6 (0.4; **0.7**; 1.5)	0.7 ± 0.3 (0.3; **0.4**; 1.1)	0.2853
hs-CRP (mg/L)	0.5 ± 0.3 (0.1; **0.4**; 1.0)	0.4 ± 0.2 (0.2; **0.4**; 0.8)	0.1445

Values shown as (1st quartile, median, 3rd quartile), [1] Total SOD activity measured in erythrocyte lysate.
